# Women’s experiences of participation in mass participation sport events

**DOI:** 10.3389/fpsyg.2022.1027285

**Published:** 2022-10-28

**Authors:** Mona Mirehie

**Affiliations:** Department of Tourism, Event and Sport Management, Indiana University—Purdue University Indianapolis, Indianapolis, IN, United States

**Keywords:** power, fear, sexual assault, sport identity, gender, small-scale sport events

## Abstract

Mass participation sport events (MPSEs) have become a popular form of recreational sport participation. Understanding experiences of participants is pivotal to designing and implementing socially just and sustainable events. Applying constructivist grounded theory methodology, this inquiry explored experiences of participation in MPSEs, with particular attention to the impact of gender on participation experiences. In-depth interviews were conducted with 13 women who participated in MPSEs. Fear and power were two core themes in interviewees’ experiences. Fear of sexual assault, injury, and “something bad” was a significant element in women’s experiences which related to perceptions of place and time. The second theme, power, was generated from strong sport identities, camaraderie among female runners, all-women races, and finishing mixed-gender races. The findings provide some guidelines for practitioners to enhance experiences of female participants and encourage repeated participation that is key to sustainability of the events.

## Introduction

There has been a tremendous growth in the number and popularity of mass participation sport events (MPSEs) over the past couple of decades (Andersen, 2021). Parallel with such growth, a line of inquiry has been developed within the sport management scholarship to investigate different aspects of participation experiences (e.g., Zhou and Kaplanidou, 2018; Piper et al., 2022). We know from previous research that experiences are subjective and within groups of people that partake in the same event at the same time, individual experiences vary based on personal perceptions of activities, other people, and places (Ooi, 2005; Volo, 2009).

Research has revealed numerous personal, psychological, and social variables that impact such perceptions, among which gender has been found to be highly influential in the context of sport events (Deem, 1987; Farrell et al., 2011). MPSEs are usually gendered spaces wherein majority of the participants are men and women tend to be enablers and facilitators of men’s participation (Raisborough, 2006; Murphy et al., 2015) rather than participators. An exception to this is the long-distance running events in which the number of female runners has had a continued increase over the past decade (Funk et al., 2011). In 2016, 61% of US road race runners were women (Running USA, 2020). While this number indicates some positive change, gender disparity remains significant in other sports such as cycling; in 2021, only 28% of US cyclists were women compared to 72% men (Zippia, 2022). Also, even those women who get opportunities to participate experience the events different from men, mostly due to traditional beliefs about genders and socially ascribed gender roles (Deem, 1987). Therefore, to establish all-inclusive events that create positive experiences and encourage continued participation, it is important to understand gender-based differences in participants’ experiences and the meaning thereof in overall life experiences (Roster, 2007).

In the recent years, European researchers have paid some attention to gender as one of the variables in studying MPSEs. Interestingly, the predominant approach in these studies was the traditional gender comparison and most of them assessed the differences in motivation for participation (e.g., Waśkiewicz et al., 2019; León-Guereño et al., 2020). To this day, an in-depth analysis of gendered experiences of MPSEs does not exist. Therefore, the purpose of this study was to address this gap in the literature by exploring women’s experiences of participation in MPSEs. Particular attention was paid to emotions, perceptions of the surroundings and incidents, and the meaning of MPSE experiences in women’s broader life context. Acquiring an understanding of such emotions and perceptions as creators of experiences is a primary step toward eliminating the negative influencers and reinforcing the positives. The subsequent enhanced experiences urge continued participation which contributes to women’s overall wellbeing, sustainability of events, and enrichment of communities.

## Literature review

Amid growth in popularity of participation in MPSEs as a recreational activity, researchers have investigated various aspects of participation such as positive outcomes (e.g., Theodorakis et al., 2015; Sato et al., 2014; Filo and Coghlan, 2016), motivation and travel behavior (Hallmann and Breuer, 2010; Funk et al., 2011; Aicher et al., 2015; Buning and Gibson, 2016), event image and meaning for locals and tourists (Hallmann et al., 2010; Kaplanidou and Vogt, 2010), event service quality and satisfaction (Theodorakis et al., 2015), involvement (Funk et al., 2011; Sato et al., 2014), and exercise intention (Funk et al., 2011).

It has been found that MPSEs can create positive attitudes toward physical activity and foster participation outside of the event contexts (Funk et al., 2011) and thus hold great potential for promoting mental, physical, and social wellbeing (Shipway and Holloway, 2010; Sato et al., 2014). Also, participation has been found to be associated with enhanced wellbeing/quality of life since positive event experiences accumulate over time and create a better overall life experience (Sato et al., 2014). Shipway (2012) highlighted the social benefits of long-distance running such as opportunities for developing new relationships and solidifying the already existing ones. Later, Filo and Coghlan (2016) applied a positive psychological lens to study charity MPSEs and found that a positive event experience creates positive emotions, positive relationships, a sense of accomplishment and meaning, and possibly a flow experience (Filo and Coghlan, 2016) all of which contribute to individuals’ overall wellbeing.

Closely related to the present study, Nikolaidis et al. (2019) assessed the impact of gender on motivation for participation in MPSEs and found that goal achievement was a strong motive for female runners, and also women rated psychological coping and self-esteem higher than men. Waśkiewicz et al. (2019) and León-Guereño et al. (2020) found similar results among Polish marathoners as women placed high importance on psychological coping and life meaning. However, men rated competition and goal achievement significantly higher than women which was different from what was found for the Greek runners in Nikolaidis et al.’s study. Although these studies as well as numerous leisure studies have evidenced gender-based differences in sport related behavior, experiences, and participation outcomes, a gender-aware approach that takes a deep look into the role of gender and unfolds the hidden intricacies of MPSE experiences has not received the attention it deserves yet.

### Women, sport, and mass participation sport events

Early work of sport sociologists acknowledged that access to sports and participation patterns differ between men and women, and therefore, knowledge about decision making processes, experiences, and outcomes is not transferable from one gender to another [I do acknowledge the existence of genders along a continuum. The binary gender divide used here was solely for the sake of convenience and clarity since all the interviewees were cisgender (i.e., born and identified as female).] (e.g., Hargreaves, 1994; Henderson, 1994). Deem (1987) discussed that in general, women’s access to sport is limited compared to men and beyond that they have less access in certain places, at certain times, and with certain partners. Location and transportation, time, money, lack of education, and societal believes about appropriateness of sport for female bodies were found to constrain women’s participation (Deem, 1987). Later, Coakley and White (1992) found that young women did not see as much value in sport as their male counterparts did. Also, parents, brothers, and even boyfriends restrained girls’ participation. Despite changes in attitudes and trends in behavior over the years, a stream of research on this topic denotes that there is still a long way to reaching a complete even ground for genders in this space. Scholars have particularly discussed that certain types of sport (e.g., snow sports) emerged as men’s domain and have remained as such throughout time (e.g., Sisjord, 2013); hence, women’s involvement is more limited and usually mediated by men (Stoddart, 2010). Even if the opportunities for initial involvement are not gender-based, women tend to be viewed as less competent, and they do not feel welcomed (Thorpe, 2009; Russell and Lemon, 2012) and therefore have higher rates of drop out (Mirehie and Gibson, 2020b).

Gendered relations of gendered societies not only directly limit women’s sport experiences but also indirectly through creating a fear of places, activities, and times that are perceived as unsafe (Sheffield, 1987; Valentine, 1989; Wilson and Little, 2008). Valentine (1989) attributed this fear to socially constructed vulnerable female identities that are deemed unable to navigate life without men’s protection. Sheffield (1987), as a radical feminist, argued that the foundation of women’s fear and gender-based power relations within human societies is sexual assault. Accordingly, men use sexual assault to terrorize and control women (Sheffield, 1987).

Previous studies on women’s leisure have evidenced the existence of such fear in leisure spaces, and it is usually heightened when women are alone or in unfamiliar places (e.g., Jordan and Gibson, 2005; Wilson and Little, 2008). Nonetheless, it has also been found that this fear is not completely debilitating. Women who have the resources and the urge to participate in leisure secure their participation by using strategies such as avoiding eye contact with men, not going out during dark hours, wearing conservative cloth, and carrying a cellphone to seek help if necessary (Wesely and Gaarder, 2004). Here, it is worth noting that from a sociological perspective, such overpreparing and planning indicates an implicit type of social control that forces women into policing their own behavior to ensure their safety, or otherwise be blamed for not acting wise (Wesely and Gaarder, 2004).

Valentine (1989) discussed that the old tradition of victim blaming that condemns women for getting violated when they were in unsafe places results in transferal of fear from potential attackers to certain public places. Subsequently, women develop mental maps of safe/unsafe places that dictate their choice of places. In other words, “they seek to escape from one form of social control (assault/rape/harassment) by exchanging it for another (surveillance and limited mobility)” (Wesely and Gaarder, 2004, p. 657).

On the contrary, women who manage to overcome their fears and endure their participation usually associate their experiences with a sense of empowerment (Deem, 1987; Henderson, 1996). Particularly, the opportunities that sport provides for physical and mental challenge (Shaw et al., 1995; Henderson, 1996), new conceptions of female bodies and physical abilities (Theberge, 1985; Myers, 2010), and development of strong identities as alternatives for the traditionally ascribed gendered identities and roles have been found to be empowering (Kleiber and Kane, 1984; Spowart et al., 2008; Mirehie and Gibson, 2020a).

As apparent in the review of the literature, women’s experiences of participation in sport as a male-dominated domain involve various contradictions and complexities. Evidently, women strive to navigate their fears, some succeed to hold their place and consequently feel empowered, some fail and end up living “like victims, within the walls of their invisible prisons.” (Madriz, 1997, p. 93). Examining women’s experiences in MPSEs as a popular contemporary form of recreational sport participation adds novel gender considerations to the literature on women’s sport participation. Therefore, this study explored how women experience activities, places, and other people within MPSE contexts. Experiences were considered holistically to include training for the event, duration of the event, and immediately after the finish line.

## Methodology

I took on a social constructionism paradigm which is underpinned by pragmatist epistemology and interpretivist ontology. Accordingly, all knowledge is local, negotiated, and constructed within the boundaries of place and time (Raskin, 2002); hence, every knowledge is only meaningful within a specific context (Raskin, 2002). Consonant with this paradigmatic standpoint, constructivist grounded theory methods (Charmaz, 2006) were utilized to conduct an inductive qualitative investigation.

### Data collection

Data collection was conducted over two summers, 2019 and 2020. In-depth semi-structured interviews were conducted with individuals who took part in MPSEs. Interviewees were recruited through purposive and snowball sampling. Individuals who were 18 years or older, and had participated in at least one race, one year prior to the data collection were invited to participate in the study. To create the primary snowball, sport clubs in a mid-west state in the United States were asked to send a digital flyer that contained information about the study to their members. Also, hard copy flyers were posted in public spaces such as coffee shops and residential buildings. In addition, a few of the researchers’ acquaintances who regularly participate in MPSEs were interviewed. Then, this purposive sample referred the researchers to their acquaintances who also participated in MPSEs, and thus, the snowball got larger and larger.

An interview guide (Table 1) was created based on the previous literature to set a skeleton for the interviews. Participants were asked to talk about the history of their participation, their motivation for taking part in events, their perception of risk and safety, their social relations within the event/activity space, and the influence of gender on their participation experiences. Adequate probing was used when more in-depth information was needed on a significant statement. Also, when necessary, the researcher provided relevant examples and further explanation on the questions to lead the construction of knowledge (Charmaz, 2006). The average duration of the interviews was 60 min. Data collection was concluded when the identified themes seemed to be saturated and no new information was impending. Interviews were conducted in-person, via phone, or video calls, recorded, and transcribed for the analysis.

**TABLE 1:** Interview guide.

What sport events do you participate in? What skill level do you consider yourself? Tell me about how you start participating in MPSEs Do you identify as a runner/cyclist/etc.? Is that an important part of your identity? **Motivation - Meaning** What meaning does the activity have for you? (Why do you like it?) What are your reasons for participating in MPSEs? Why? What are the benefits that you get from participating in MPSEs? Any costs or negative outcomes? How do you cope with the negative outcomes? What are your overall attitudes toward the community events and why? What other emotions do you have of participation in MPSEs? **Social Dynamics/Family/Friends** Who are your ————– buddies? What part do your buddies play in your experience? Are there differences between when you participate with different people? Do you ever participate in MPSEs alone? **Risk** Do you think there are any risks associated with repeated participation in MPSEs? How do you manage those risks? Does participating with your buddies make any changes in your perception? **Gender** Does your gender impact your experiences at events? Have you noticed any gendered dynamic/behavior? Does your gender impact your perceptions of yourself as a runner/cyclist/etc.? Have you ever participated in women-only races? How was your experience different from mixed-gender races?W8748

### Data analysis

As per the constructivist grounded theory procedures (Charmaz, 2006), the analysis aimed to identify patterns in the “subjective and collective experiences” (Charmaz, 2008, p. 204) of a social phenomenon (herein gendered experiences of MPSEs), discover the links among theoretical concepts, contextualize such links within broader structures, and develop frameworks that explain the phenomenon under examination.

**FIGURE 1:**
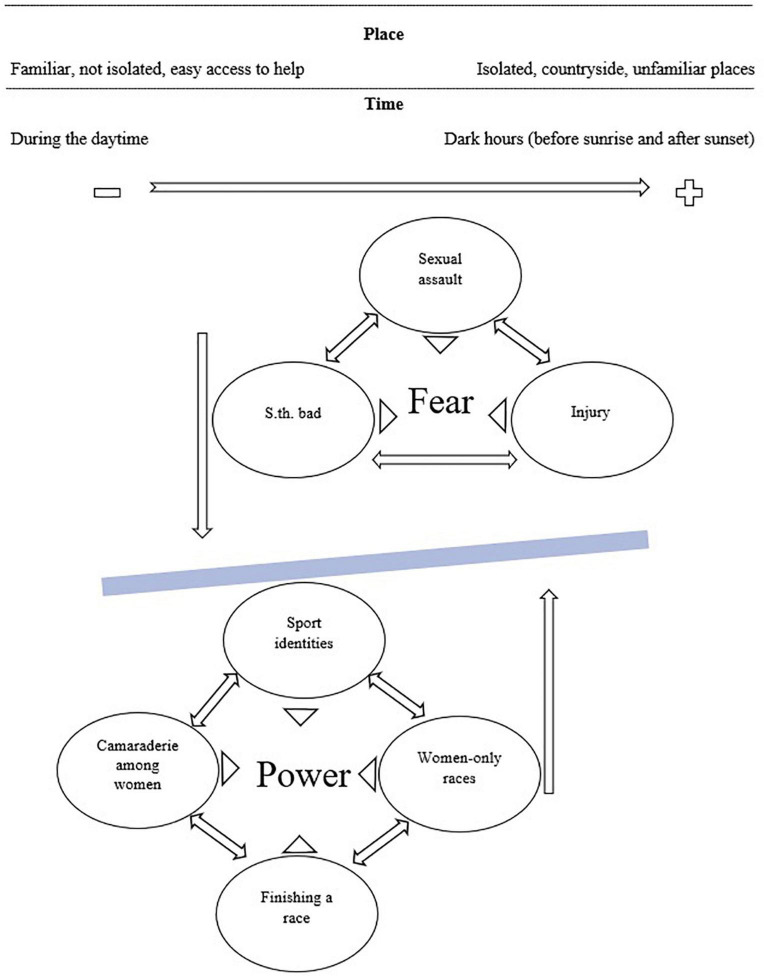
Grounded theory framework of women’s fear and power within MPSEs.

Following Charmaz’s (2006) guidelines, data analysis was done at the same time with data collection. Constant comparison methods (Glaser and Strauss, 1967) were used to revise and refine the interview questions, codes, and interpretations throughout the process. The analysis was started by incident-to-incident open coding. Then, the most frequent and prominent codes were used to guide the focused coding. Next, axial coding was applied to find the relationships among the codes. Finally, through theoretical coding, a framework was developed that presented the relationships among the main concepts in gender-specific aspects of women’s experiences of participation in MPSEs (Figure 1). To check the credibility of the analysis, the interviewees were asked to review and validate the write-up of the findings (i.e., member checking).

### Participants

Initially, twenty-three individuals (*N* = 23; Women *n* = 13, Men *n* = 10) who participated in MPSEs were interviewed. This paper only presents data from female participants. Interviewees’ age ranged from 27 to 61 years old; the largest proportion were in their 30s (*n* = 6). The level of education ranged from undergraduate to doctoral degree. Most of the interviewees were married (*n* = 8) with children (*n* = 7). All interviewees noted running as their primary sport, however, stated that they participate in different types of events such as marathons, biathlons, and triathlons. Also, all indicated that they belong to a running club or group and skill level ranged from amateur to advanced (Table 2).

**TABLE 2:** Profile of the interviewees.

Pseudonym	Age	Marital status	Children	Education	Sport	Skill level
Maya	32	Married	2	Bachelor’s	Running	Amateur
Kim	27	Single	0	Master’s	Running	Advance
Hannah	61	Married	0	Bachelor’s	Running	Upper-intermediate
Lena	56	Single	2	Bachelor’s	Running	Advance
Lydia	36	Married	2	Bachelor’s	Running	Intermediate
Cara	37	Married	1	Master’s	Running	Intermediate
Tara	32	Married	0	PhD	Running	Intermediate
Tina	44	Married	2	Bachelor’s	Running	Advance
Jas	44	Single	0	PhD	Running	Intermediate
Ryana	39	Divorced	0	PhD	Running	Intermediate
Kayla	52	Married	2	Master’s	Running	Intermediate
Leslie	30	Single	0	PhD	Running	Intermediate
Paula	55	Married	2	Master’s	Running	Amateur

### Findings

The analysis revealed commonalities in experiences of men and women; however, two themes uniquely appeared in women’s narratives, power and fear. Power had four associated subthemes; sport identities, finishing a mixed-gender race as a woman, camaraderie and support among women, and women-only races were perceived as empowering by women. On the flip side, a fear factor was evident in experiences of all women that had three subthemes: sexual assault, “something bad happening,” and injury (Table 3).

**TABLE 3:** Themes and subthemes identified in the interviewees’ narratives.

Theme	Subtheme	Indicators
Power	Sport identities	[sport] is — —my thing —my personal time —my getaway —an important part of who I am —an alternative to family/work identities —a part of being a whole person —s.th to be proud of outside of other things —what makes me a well-rounded person
	Camaraderie among female runners	Being with like-minded people Bonding over sharing personal stories Having a sense of community Having a good social outing Being less serious about the sport Being encouraging and supportive Having a sense of belonging Being able to start new friendships
	All-women races	Centering everything on women Everything is about empowering women Focusing on women’s victory Women cheering for each other Celebrating women’s wholeness Being comfortable for beginners Being welcoming Getting camaraderie with women
	Finishing mixed-gender races	Getting over self-doubts Keeping up with men Beating men (who are bigger and faster) Boosting self-esteem Crossing the finish line Humbling men by winning Feeling accomplished Getting over intimidation of men
Fear	Sexual assault	Hearing about sexual assault on media Being afraid of getting attacked by men Talking about assault with other women Reading about assault on running blogs Staying alert when running Using safety precautions (e.g., not wearing headphones when running) Staying on familiar routes Being catcalled while running
		Running on well-lit routes Being afraid of rape Being warned by male relatives
	Injury	Being afraid of — — physical injury — twisted ankles/knees — tripping and hurting yourself — medical emergency or passing out
	“Something bad”	Being afraid of something bad happening Not knowing what is going to happen Being afraid in the first two miles of races Fearing being in a bad situation

### Power

A prominent theme in interviewees’ statements was power. Maya said, “It just makes me feel good. I just feel active. Powerful keeps coming in my mind.” Factors such as identities constructed through sport (e.g., runner and cyclist), finishing a mix-gender race as a woman, camaraderie and support from other women, and all-women races in which everything is centered on women were perceived as empowering for female runners.

#### Sport identity

Identities that women constructed through their sport were found to be highly empowering to women as they were considered to be alternatives to traditional feminine social identities of mothers, daughters, wives, sisters, etc. that are associated with domestic gender roles. This became evident when interviewees referred to their participation as “my thing,” “my personal time,” and “getaway.” Maya expressed it very clearly:

It took a while to identify as a runner officially. It feels good now. I feel good about myself because it’s my own personal thing regardless of anything, family or anything that’s going on. It is always there, and I can go out and run and feel powerful.

Kara explained, “I had a baby in December, and I planned to do marathons in April. It gives me that one thing that is still mine, that time to myself.” Similarly, Ryana said, “I think it’s just the time where I can spend with myself and with my thoughts. Any other time in my day I usually don’t have that luxury.” Tara articulated:

Being a physically active person is a really important part of my identity and being outdoors is a really important part of who I am. If I got injured to the point that I could not run, I would do something else that still keeps me active and outside. It happens to be running now, it could be cycling, it could be rock climbing, or skiing.

She also associated the challenge of physical activity with internal satisfaction, sense of accomplishment, strength, and good relationships:

I really enjoy it, like I want to be outside all the time so anything that gets me outside is great. I find very demanding physical activity to be extremely satisfying. It is a really strong sense of accomplishment; it makes me feel strong. It gives me opportunities to connect with other people who also like those things, so it helps with my relationships.

Ryana also mentioned:

When you go through many events and you just kind of feel better after a run no matter what happens in your life, you start realizing that this is a very important part of who I am, and a part of my mental health, and part of just like my happiness in life so because of that I continue.

When Tara further elaborated, it became apparent that for her being physically active and outdoorsy was a way of pushing against the traditionally ascribed gender expectations:

I feel like my gender has been a barrier because I’ve always felt like guys are expected to enjoy the great outdoors and women are kind of cool or different if we like the outdoors… I run with a lot of people that have low expectations of me like I’m not capable to do things… It was important to me when I was younger to prove people wrong and show them how strong I am.

Lena constructed her new self through running after she got a divorce in her middle-ages, she said:

It was a life-changing event for me as of somebody entering middle-age. I was deciding to get divorce and I got divorced, and on these races, I was entirely alone. I was totally self-motivated. I had very little like “your mom” or “your wife.” I was alone. I remember crossing the finish line and thinking I am alone, this is for me, I’ve achieved this by myself with my own fitness and my own determination.

Later, she added, “That’s part of my life that’s important. That’s an important part of just being a whole person… It’s something that gives me some pride in myself.” Lydia clearly called her sport identity an alternative to her family and work identities:

It’s my time. I have two children and I’m a teacher, so I spend all day taking care of other people’s children and then I come home and take care of my own children again. For the hour or whatever, that’s my time, it’s my time to think about things that are bothering me or things that I’m happy about.

She noted the opportunity that running provided for her to stretch her limits which mirrored the sport literature that highlights the opportunities for physical and mental challenge that help women to recognize their abilities and build strong alternative identities:

It’s nice to say I’m going to do this and then to do it and then take it one step further. It gives me a sense of accomplishment and it helps stretch me further than I thought I could go… until you push it you don’t know what you’re capable of. There’s a lot of accomplishment in that end that’s motivating me to go a little bit further. That’s why it gives me my time to be Lydia instead of mom or Ms. Smith. It gives me something to be proud of outside of the other things that I do.

Leslie also explained that overcoming the challenge makes her feel empowered:

I want the challenge. I want to see I can run a marathon. It’s a pretty hard thing to do and if I can do it then I have that sense of I’m powerful, my body is strong, and so that’s really invigorating and it’s like if the medal looks cool, the swag is really neat, it’s like I could run this on my own or I could pay whatever till I can get the cool stuff that I’m going to wear and I’m gonna identify as a runner.

Cara’s statement further emphasized this, “Strong, dedicated, and disciplined define who I am, and I do it through running.” Leslie referred to running as “another part of me,” she said:

It makes my life more fulfilled or more purposeful in some way. I have a job that brings me fulfillment, but this gives me a way to develop another part of me and another part of life that’s more fun. There’s a well-being aspect that I wouldn’t get from teaching or being a sister or daughter.

Kayla briefly said, “It is an important part of my identity … I think it’s a great metaphor of life… I never believed in myself as an all right confident … seeing my progress makes me feel more confident.” Tina considered her sport identity to be complementary to her other identities that all together make her a well-rounded woman:

It isn’t the only part that defines me but it’s one place that I have control over my success, or at least the effort that I put in. I balance that with community service and engagement with my work. I feel well-rounded like I’m not defined by a single undertaking.

Lena said, “I am really trying to eliminate the stressors caused by my identity as a mother and ex-wife. I am just trying to be in the moment, I don’t think about anything except just completing this race.”

#### Finishing a mixed-gender race as a female runner

Another factor that women considered empowering was finishing mixed-gender races. Most of the women indicated that while they did not necessarily compete with men, pushing their own limits, overcoming their fears, and finishing a race made them feel powerful. Maya stated, “I second guess myself a lot but at the end of the day it doesn’t really matter. Although it’s in my mind at the beginning, at the end of the run I am not thinking about it anymore.” When asked to provide further explanation, she elaborated:

There have been times where I have been among a lot of men and I felt intimidated… Men are faster, men are bigger… at the end of the run I’m like remember how you were worried about that? you still did it anyways. that makes me feel powerful in the long run.

Similarly, Ryana noted that she does not care about competition, but she likes the challenge of racing with men who are “bigger” and winning over them feels good to her:

I don’t need to beat somebody, I run for myself but when you’re in a race and there is somebody’s butt in front of you, you want to beat that. It really does motivate you when you see other people going through this. I did a trail race that was very hilly, and you needed to make a lot of tiny steps not to be clumsy and I beat so many men in that race because they are bigger and less agile. It pushes you a little farther than you typically would go… I get super excited, I guess.

Explaining this further, she again emphasized that men are “bigger” and thus beating them in a race feels good to her, “In races, that male competition might motivate you. I see myself as a much weaker runner than men. When you beat somebody who’s much stronger you get a boost of self-esteem. Those guys look big, can probably lift a truck.” Lydia had similar opinions, however, stated it more implicitly:

I tend to stick myself up there with all the men. When you’re getting ready to start a race and it’s all men, I see that as motivation and I say, I can’t catch them but if I can at least keep them in my line of sight then I’m doing okay. I find it motivating to see many men up toward the front because that makes me think I could be there with them.

Lena also highlighted that crossing the finish line gives her a sense of self-confidence and power that she does not have otherwise:

Many times, when I cross the finish line… um, it was a 10K it was a very hilly race that was quite a challenge for me and my fitness at that time. I was by myself, and I really felt like Lena this is your gift to you, you’re crossing the finish line. I think symbolically, I think metaphorically it was a life-changing moment when I said I am going to try to go forth by myself. I am paddling my own canoe here, I’m doing it. Because of the fitness and the running, I gained a sense of self-confidence I never had before as a woman.

She paused and seemed to be deep in thought for a minute, then continued, “a sense of power, a sense of confidence, pride in myself that I can! I’m not telling everybody, I’m telling my inner child, my inner person, my inside, look what you did. You did it again.”

Tina stated:

It’s empowering to be able to pass a guy or to hang on to run with him, to be as capable, we’re not the back half, we’re all mixed in. It is challenging as a positive. I don’t feel diminished because they’re there and I hope it’s humbling for them when we pass them.

Similarly, Leslie explained:

It is a challenge. I’m on the course and I see a bunch of guys around me, it’s an ego boost because guys are faster. It’s like oh, I’m not doing bad… when I see that I can run with guys it is a bit of encouragement and ego boost… I guess being able to run in a pack with guys just kind of makes me feel empowered. Guys are faster; I feel accomplished.

For Maya, competition was not an important factor, rather overcoming her own self-doubts and fears made her feel empowered, “I don’t let the fear stop me. By the end of the run, I don’t think of it anymore. It makes me feel powerful.”

#### Camaraderie and support among women

Another aspect of participation that created a sense of power for women was camaraderie and support among female runners. Kim briefly mentioned, “I think it’s more of being able to be with people who can share the same feeling or motivation.” Ryana further explained, “On a regular basis I run only with women. I feel a little insecure, I tried to run a couple of times with a guy from work and he needed to go super-fast. I didn’t enjoy the experience.” When asked to talk about what makes a good experience, she explained how women bond over sharing stories when running together, she said, “We share very personal stories. There is a rule that what you talk about in a run, you don’t share outside a run. We have that sense of community… I have made a lot of good friends through runs.” Cara also noted the talks:

Long-distance running is not an aerobic activity, you run 10-12 miles but still you can talk, and you can talk about everything, your job, your life, your relationships, your marriage. I mean the group of women are known for their accountability and their support for running. We share experiences. If there is good staff, we celebrate, when someone gets married, we celebrate by a runaway bride party in the 5K, we do baby showers. It is a very unifying sport.

Maya stated that such camaraderie makes her feel empowered, “I have met a lot of female runners that are involved with the events that I care about. We run, walk, and do different exercises, and that feels really good as a social outing, and I feel powerful afterwards.” Tara further elaborated:

When I run with women it tends to be less intense about the pace and distance. I don’t feel like they spend the whole time trying to get me to run faster or trying to get me to run farther. When I run with men it just feels like what I do is never good enough, and when I run with women and they’re so supportive and so encouraging and everyone’s so proud of each other and it’s just such a good feeling.

Similarly, Tina said:

A lot of my friend circle and connections are in the running community. It’s a sense of belonging. It helps me add deeper connections with my friends or at least a starting point for friendships, and then we can get to know each other outside of that. That’s a place that I have a good level of dedication and I don’t want to sacrifice, I’m protective of it. It makes me feel good, I feel stronger and healthier, and they connect me with other people and that’s important.

Jas briefly said, “Having that group of women that support each other rather than women that tend to let each other down is empowering in itself.” Likewise, when asked why running with other women is empowering, Leslie stated:

We all care about each other as people. Running is just something we have in common that gets us together. When we’re on a run we’re not talking about the run, we’re talking about our families or classes. Everyone is really supportive and encouraging.

Hannah explained, “Everyone is cheering for everybody else, there’s no negativity. I’ve made great friends… You’re out there for a couple of hours, you talk about everything, your family, your marriage, your work, you share so much.”

#### Women-only races

Some of the interviewees that had participated in women-only races perceived those to be particularly empowering because everything (e.g., the event theme, the swag) is centered on women. Hannah said, “There are events that are kind of themed around women and empowering women. I like the women-only theme … you get that camaraderie right across with women …the shirts and designs are targeted towards women …they’re all about empowering women.” Likewise, Tina who participated in women-only races on a regular basis said, “I think there’s is more of a sense of camaraderie, women supporting women, and a lot more like encouragement from participants to each other.” When asked to explain this further, she articulated:

Just the little comments, good job! or you stick with me. The way that women dress up, somebody had a bridal veil they were getting married. People cheer you or make comments to support each other that way … The finish line especially, knowing like a woman won, there’s no distraction from her victory, there’s no guy that broke the tape ahead of her, oh yeah you make space to celebrate your wholeness.

Cara found the women-only races as a comfortable space for the beginners, “I just think it’s maybe a more comfortable place to make their [first timers] debut because they’re surrounded by women.” Maya explained, “It feels good. It just makes you feel a little bit better. They welcomed me, I was able to do, it I can do more next time and then that just carries on.” Hannah said, “There’s one [event themed around women] here that they were going to stop. I cried because I love it. They relented and are going to run it again next year.” When asked why she loves it, she explained:

The women-only events are different in that you get that camaraderie right across with women. They market it to women. Everything about it is centered on women … like one serves cake! What event serves cake?! One had flip-flops and sunglasses made up around the theme of the race. They’re all about empowering women.

Tara said, “If I went to a women-only event I would be much more comfortable taking off my shirt and running in my sport bra if it was too hot or I wouldn’t even think about that I would just do it and I wouldn’t be fighting against the guys for the first place. I usually have a really great time with women on trails.”

### Fear

Another theme in women’s narratives was fear which appeared to be related to vulnerable female identities and women’s lack of confidence in their own physical abilities to either finish the race or protect themselves in case of danger. Sense of fear was expressed as “anxiety,” “nervousness,” or “being scared,” and it was linked to women’s perceptions of place and time. When asked to talk about her emotions during a race, Ryana said, “Definitely a little bit of nervousness. I do not see myself by no means a competitive runner or a super-fast runner.” Although numerous times during the interview she mentioned that she runs for herself and does not care about competing with other people, not being a “competitive or super-fast” runner still made her nervous. Such self-doubt appeared in Leslie’s statement too, “I get nervous. Am I going to be able to finish?” Maya stated that part of her fear/anxiety is created by male relatives, “When I run alone primarily the men in my life get concerned about my safety. Like my dad or my uncle.” Similarly, Lena said:

I stay on the sidewalks and there’s people coming and going and I’m running. It’s very public. But [partner] is like baby take care of yourself, don’t park in this place cause you have to go down that lonely stretch to get back. Park by the stadium, it’s more public. You must be wise. Unfortunately, the world is not an even place, you must plan ahead.

It became apparent that she always saw herself in need of her partner’s protection, “With [my partner] I’m protected … he’s really strong so I feel good, I feel comfortable with him.” Likewise, Cara mentioned protection from her male running partners:

They’re a little bit protective. I’m imagining one relay race where you ran in the middle of the night. We were on this country road, and so I didn’t even know this person, but he and I were running at the same time, and he specifically said I’m going to run on the outside.

#### Sexual assault

The most salient fear factor in women’s narratives was sexual assault even though none of the interviewees had experienced assault at any races. It was found that this fear was mostly socially created, by either male relatives or runners. Maya said that men in her family get worried whenever she goes for runs, then she explained, “You hear like sexual assault more often happens to women. You hear stories about a woman was running and got assaulted. I think they [her male relatives] hear those things, and they think it happens more than it does.” Hannah said, “We talk about it. I’d bring one of the women in our group because you know people have been assaulted when they were running alone … It’s something people are very aware of.” Talking about fear of sexual assault, Cara also thought she needs to be alert and aware of her surroundings all the time:

I think about it … again it depends on where you run. I don’t put headphones on. It’s important to be aware of your surroundings, things that make you more vulnerable like if you could not hear people around you, if you don’t know that someone is there. There are choices that I make to be safer, again a big part of it is knowing your routes.

Likewise, Lydia explained, “I keep it in my mind to be aware of my surroundings. I don’t wear headphones when I’m running. I try to stay aware of my surroundings and to not be alone in a secluded area.” Lena’s fear not only affected her choice of place for running but also her choice of clothing, she articulated:

As a woman running you must be really wise about where you’re going … When it’s really hot outside and I’m stuck wearing a sticky shirt, could I run in my sports bra? that would be amazing, but I won’t … I feel like that would make other people uncomfortable. It would send a weird message to men.

Tara elaborated:

When you’re out there by yourself and it’s a place that people might not come by for a while or the places that are easy to walk to from town you just don’t know who’s gonna be out there and you don’t know if there’ll be cell service or if you can call emergency services if you need to. Really, it’s an issue of being afraid of being attacked by men. I’m not really worried about animals or getting injured out there, that doesn’t scare me at all. For me it is entirely coming across dangerous men.

Ryana also noted hearing stories that make her feel uncomfortable in certain places and she preferred to run around people and where she can easily manage her route:

I am scared of being by myself on a trail because on some runner blogs you read stories like woman got raped, or how this guy was trying to shock the lady, you do feel kind of weird being by yourself in the woods. I mostly run during the daylight if I run by myself, I only go on trails that I have well-traveled, somewhere in the park rather than running in a forest where nobody runs. When I run in town there are places where if I don’t know what this person is doing but he acts kind of weird then I just run in a different direction.

She was the only interviewee who shared an actual experience of sexual harassment while running:

I had a scary situation where somebody flashed me on the run. I was running and this guy kind of turned my way and pulled down his pants. I was very careful not to see any details. I stopped for half of a second and then I sprinted towards him, and I did not expect that from me, but I was so mad. I was like I’m gonna beat your [curse word]. He ran away from me.

Leslie brought up catcalling at female runners, “You know comments that people yell out of their cars or whatever as you’re running. Unnecessarily annoying.” She further explained:

Being in a new place or somewhere I’m unfamiliar with and then just having kind of that dead space of like the starting line or like the after party or whatever especially if I’m not traveling with anyone and it’s just me at it then that would certainly be a time where I would be more concerned about that [potential assault].

Lydia added, “Like you’re running down the road and some jerk in a car honks at you and whistles out the window. It’s always in the back of my head to be aware and watch out for things that seem off.” Cara also said, “A lot of people yell at people when they are running. ‘Oh, look at you girl’ those are things that make me feel more uncomfortable than encouraged.” Lena shared a similar experience:

There was a weirdly sexual thing one time. It was an old man told me I had a good stride that was long. Then there was this other man who came to me after the race and said you were competing with me. I was alone. I was very tired, I wasn’t looking or competing with anybody I just want to survive.

Paula noted that she gets scared when it’s dark and that with her running partners they are always alert:

It’s because it’s dark and there aren’t many people around. Even with the group or with one partner we still are very ware of our surroundings. We make sure that we have good lighting. We don’t run in the bushes or anything like that unless it’s daytime.

When asked to explain what she was afraid of, she said, “It’s the attacker, it’s rape, that kind of thing. It’s very rare. We had one probably six or seven years ago but it only takes one to get everybody saying I need to be careful and so we are.” Maya, again, emphasized that she was warned about sexual assault by men in her life who thought she was at risk of assault when she ran alone and in certain places like downtown, she said:

“When I run alone, primarily the men in my life like my dad will get concerned. They’ve expressed concern over where I run. When I’m downtown, I don’t know where they’re picturing me running exactly. I think because you hear about those things happening to women, got assaulted.”

Similarly, Kim talked about hearing stories about assault that make her anxious, she stated, “I definitely feel like women are at risk more than men and then because I always watch the news. Like last week someone got attacked because she was running alone.” Hanna also said, “People know about people who got attacked when they were running alone. I do not hear men talking about it. It is a gender thing.”

#### Something bad happening

Interestingly, while most of the interviewees experienced fear during races, many of them were not even sure what they feared. While this was implicitly stated when women talked about always being alert, being prepared, and planning ahead without pointing out any particular fear factor, some explicitly talked about it. For example, Tina shortly said, “It feels a little scary. Something bad is going to happen.” Leslie had a similar fear of something bad, “It is a little bit nervous like coming up to the race or the starting line who knows what’s going to happen.” Paula usually started the race with fear of something happening to her, she said, “The emotion that I feel the most in the first two miles is fear. I’m always afraid that I am going to be tripped or pushed in that busy time … I’m always in fear for the first two miles.”

Cara also was scared of potentially being in a bad situation and therefore chose to run in familiar places:

I run somewhere that I know, and I am aware of it. If I’m in a bad situation, I know where to go, like I would run to a hotel lobby or home. Those are things that honestly are always going on for almost all female runners whether in the top or back of their mind.

#### Injury

Another fear factor was injury. Most of the women appeared to be anxious about possibly getting injured at the race. Paula said, “I’m afraid, the fear of the physical injuries because of the crowds at the race.” Kim said, “What if a medical emergency happened or I passed out?” Lena added, “No twisted ankles, no twisted knees because twisted ankles mean that you are out for years … So, it’s like every time I go outside to run there’s a huge risk.” Lydia also stated, “There’s always in the back of your head that you’re going to trip and hurt yourself.”

Evidently, sense of fear was mobile and strongly related to women’s perceptions of place and time, wherein an unknown course in a new race or destination, isolated areas, or the countryside created/increased the sense of fear. Leslie said she was afraid of new unknown courses, “If it’s a race I’ve never run before there’s some sense of the unknown, of what the course looks like. It is a bit nervous.” Likewise, Kim said, “I’m more conscious of where I am if I’m not doing the same races that I usually do, I’m like looking around. I don’t want to be running a place that is not safe or well-known.” Tara’s fear was also linked to her sense of place unlike other women she felt safer in isolated trails where humans are unlikely to go, she elaborated:

I get nervous if I’m on the trail by myself especially if my dog is not with me. It depends on where you live, if it was a trail that people didn’t hunt on, I feel safe, or a trail that was further removed from the city. But the trails that are right next to town or commonly hunted on, I would definitely worry about coming along a couple of guys or one man, and it being a threat to my safety, getting shot at or getting assaulted.

Ryana expressed her fear as a dislike for dark and isolated areas, “Sometimes if I go somewhere like it’s kind of dark, I don’t particularly like that, like excluded area.” Lena picked different routes when she ran without her partner, “I do run by myself sometimes. I’d be careful with what I’m picking. I’m checking my routes and what not.” She further explained that while she has not experienced sexual assault, she is careful with her choice of place when she is alone:

You must take responsibility for yourself whether you’re walking on the street, running, or taking a little stroll in the forest. I’m pretty careful to pick very public places like the [University] campus. If it’s wintertime and its dark, I don’t go around the lakes.

Similarly, Kim summed it all up, “If I do run alone, it’s during the day and it’s on a similar route and I take a phone.” Leslie also said, “If it’s just me, I’m going to be much more mindful of the time of day that I’m running, the location where I’m running. I’m gonna run where there’re cars so if something happens someone’s at least seeing it.” Hannah explained that because of her fear of sexual assault she runs during the daytime and in familiar areas, “I usually stick to areas that I know well, if I am by myself, where I feel safer. I don’t run at night. I’d rather be able to see what’s around me.” She further emphasized that such particular attention to the choice of time and place is specific to women, “It is a gender thing, I think it is a gender thing.”

Leslie tried to manage her fears by always letting her friends know when and where she runs, “Before I leave, I text a friend and say hey I’m going for a run and I’m going to run the botanical gardens or downtown or whatever, and then when I get back, I’ll text them.” Paula ensured her safety by never running alone, “I never really run alone. I always run with at least one more person and it’s also because of safety.” Tara also said,

I usually ran with people and if I’m not running with someone I tell someone where I’m going; so if I don’t come back for a long time they will know why, but if I’m in the back country running by myself, I always bring my cellphone with me so I can call emergency services if I need to, but I always run with people it makes me feel safer and is more fun.

In summary, my analysis of MPSE participation experiences revealed two gender-specific themes that only appeared in women’s accounts, power (subthemes: sport identities, finishing a mixed-gender race as a woman, camaraderie and support among women, and women-only races), and fear (subthemes: sexual assault, “something bad happening,” and injury). Power and fear seemed to act in opposite directions, but both were related to mobile emotions associated with perceptions of place and time. Fear was exacerbated during dark hours (i.e., before sunrise and after sunset), and in places that were perceived as unsafe such as countryside, and new/unfamiliar courses or destinations (Figure 1).

## Discussion

This study investigated women’s experiences of participation in MPSEs. Similar to other aspects of human life, participation in MPSEs was found to be a nuanced socio-cultural phenomenon that involves multiplex ties of traditional believes about gender and sport, feminism, socially constructed notion of safety, as well as perceptions of place and time.

The findings regarding power mirrored previous research on women’s participation in sport. Mental and physical challenges involved in sport help women fight against the expectations associated with traditionally defined gender identities such as wives, mothers, and daughters and construct sport identities as strong self-created alternatives that diverge from socially ascribed gender responsibilities (Kleiber and Kane, 1984; Spowart et al., 2008; Mirehie and Gibson, 2020a). Overcoming challenges inherent in sports has been linked to women’s empowerment which is not only an outcome of participation but also, for many women, a motive for initial involvement (e.g., Shaw et al., 1995). The present study supported previous research on empowerment through sport by highlighting the value of sport identities for women.

Interestingly, the independence of sport identities from the immediate social circle (e.g., children, husband) was highly valuable; however, the social networks around the sport appeared to be equally appreciated. Particularly, all-women sport communities and camaraderie among women were deemed highly empowering which, again, represented previous research in that all-women leisure contexts and bonding among women results in empowerment (e.g., Mirehie et al., 2018). The contribution of this study to the literature was revealing the existence of such dynamics in MPSEs. It is particularly interesting that the appreciation of so-called feminine traits and interests such as a pink theme resulted in generating a so-called masculine trait, power. Perhaps, in women’s mind, such appreciation translates into welcomeness in a space from which they have been excluded for many years, and an attempt to correct the outdated gendering of activities and places that are not gendered in essence, herein MPSEs. Indeed, this could be explained by several feminist schools of thought. For example, radical-libertarian feminists believe that humans should be free to be androgynous and show both feminine and masculine qualities (Tong, 2009). From this perspective, MPSEs could be viewed as opportunities for women to explore and integrate their feminine and masculine dimensions and experience a sense of wholeness. Alternatively, radical-cultural feminists contend that the society’s gender-based problems root in the low value placed on feminine traits (e.g., gentleness, supportiveness, and empathy) compared to masculine traits (e.g., aggressiveness, hardiness, and rationality); hence, if the feminine and masculine traits are valued equally, women’s oppression will come to an end (Tong, 2009). From this standpoint, the appreciation of girly swags/themes that became apparent in women’s narratives could be interpreted as that women perceive those as a manifestation of value placed on feminine traits and a step toward eliminating patriarchal discriminations.

The findings indicated that despite enjoying the experience and feeling empowered, women felt a fear factor in their MPSE experiences that was bound to their sense of place and time. This mirrored previous research on different domains of women’s life such as outdoor recreation (Wesely and Gaarder, 2004), solo leisure travel (Wilson and Little, 2008), and business travel (Mirehie et al., 2020). Participants’ narratives disclosed their subconscious self-doubts and acceptance of a subordinate position in the society’s gendered power hierarchies that dictate what activities, times, and places are “safe, right, and sensible” for women (Wilson and Little, 2008, p. 182). This study added to the literature by evidencing the existence of such fear in MPSEs which was particularly interesting since the primary assumption could be that MPSEs, as highly structured and organized sport settings, would generate a higher perception of safety compared to independent unstructured participation. The fact that similar results have been found in different contexts could imply that within the gendered structures of human societies, fear has become an existential element of women’s life experiences. Fear of physical injury was another attestation to this point. Although this was a weaker theme compared to other fear factors, it was gender-specific and only noted by women. Also, many women could not even point out what they feared which was expressed as fear of “something bad happening.” Merely, by virtue of being a woman, they constantly feared a potential negative encounter or situation and a need for being careful.

Sexual assault was found to be the most prominent fear factor in women’s narratives which resonated with previous research on women’s fear (e.g., Baumer, 1978; Balkin, 1979; Gordon et al., 1980; Warr, 1985; Sheffield, 1987). Sheffield’s (1987), in her theory of sexual terrorism, recognized sexual assault as the foundation of women’s oppression, gender-based power relations, and all discriminations. Although this study was not conducted from a radical feminist standpoint, I acknowledge the relevance of Sheffield’s theory and that it could act as one possible explanation of my findings about terror of sexual assault.

Furthermore, fear was found to be closely related to perceptions of place and time which reflected the core ideas of cultural and emotional geographies (Valentine, 1989; Bondi et al., 2005) that discuss constructed notions of safety or lack thereof. Correspondingly, women socially learn to be constantly fearful and cautious about their surroundings in certain places and at certain times that are deemed unsafe (Wilson and Little, 2008). The process of social construction was apparent when participants talked about hearing about sexual assault and getting concerned about their own safety. Or the fact that the men in their lives like father and/or husband worry them and warn them about sexual crime. Such warnings caused them to believe they are vulnerable and at risk in most circumstances and to doubt their ability to defend themselves in case of an accident (Valentine, 1989). So, per this socialization and constructed perceptions of safety, when it comes to training, women undergo a process of self-surveillance by setting spatial and temporal restrictions on their use of public space or alternating their choice of clothing (Wesely and Gaarder, 2004). In a MPSE setting, the course is set and cannot be changed; therefore, fear becomes an innate piece of women’s experiences.

Although the first theme illustrated that participation in MPSEs is to some extent empowering to women, the fear factor indicated that power is still not equally divided. This could reflect the broader gender-based power relations within the patriarchal systems where the public space is not evenly shared (Wilson and Little, 2008), and microaggressions such as catcalling, whistling, or staring often alarm women about potential danger (Valentine, 1989). Consequently, women’s sexualized bodies are restrained by either visible male gaze or women’s own invisible self-surveillance.

## Conclusion and implications

The findings provide an understanding of the role of gender in shaping MPSE experiences which can guide practitioners to address the gender disparities in participants’ experiences. It was found that acknowledgment of women’s sport identities, feminine traits, and supportive environments created a sense of empowerment for women. Acknowledging female participants by offering girly swags or themes, appreciating women’s participation in promotional materials, and creating supportive environments could be primary steps toward opening more space for women in sport and gradual cultural change.

Fear was found to be another significant element of women’s experiences. As discussed above, a great deal of perceptions of danger and the consequent fear are socially constructed However, the existence of such feelings reveals a problem within the MPSEs and its impact on the experiences should not be neglected. Event organizers can address this issue by trying to minimize perceptions of danger through enhancing security provisions, choosing routes that are commonly perceived as safe, and increasing access to help throughout the course. Such simple amendments could help in changing the imagery of danger within MPSEs which over time could be extended to the broader society.

This study provided some insights into gender and MPSE experiences. I acknowledge that while there are similarities in human experiences based on gender, there is not a homogenous category of people called women. There are numerous other social, cultural, and economic factors that impact women’s lives. Also, this manuscript did not aim to represent a holistic view of women’s experiences of MPSEs and rather was focused on aspects of the experiences the were gender-related (i.e., distinct from men’s experiences). Hence, more research is needed to assess the role of such influencers and provide a more holistic view of this subject. Certainly, empirical investigation of men’s experiences would be another valuable addition. Last but not least, in this study, I applied a qualitative approach to delve deeply into women’s narratives, using quantitative methods to test the findings at a large scale and expand the findings could be another direction for future research.

## Data availability statement

The datasets presented in this article are not readily available because to maintain confidentiality, the author reserves the right to not share data other than the excerpts that were presented in the findings section of the manuscript. Requests to access the datasets should be directed to MM, mmirehie@iu.edu.

## Ethics statement

The studies involving human participants were reviewed and approved by Indiana University Institutional Review Board. The patients/participants provided their written informed consent to participate in this study.

## Author contributions

The author confirms being the sole contributor of this work and has approved it for publication.
